# Refinement of the assignment to the ACMG/AMP BS3 and PS3 criteria of eight *BRCA1* variants of uncertain significance by integrating available functional data with protein interaction assays

**DOI:** 10.3389/fonc.2023.1146604

**Published:** 2023-04-24

**Authors:** Laura Caleca, Paolo Radice

**Affiliations:** Unit of Predictive Medicine: Molecular Bases of Genetic Risk, Department of Experimental Oncology, Fondazione Istituti di Ricovero e Cura a Carattere Scientifico (IRCCS) Istituto Nazionale dei Tumori, Milan, Italy

**Keywords:** hereditary breast/ovarian cancer (HBOC), BRCA1, VUS, functional analyses, protein-protein interactions (PPIs), ACMG/AMP guidelines

## Abstract

The clinical screening of cancer predisposition genes has led to the identification of a large number of variants of uncertain significance (VUS). Multifactorial likelihood models that predict the odds ratio for VUS in favor or against cancer causality, have been developed, but their use is limited by the amount of necessary data, which are difficult to obtain for rare variants. The guidelines for variant interpretation of the American College of Medical Genetics and Genomics along with the Association for Molecular Pathology (ACMG/AMP) state that “well-established” functional studies provide strong support of a pathogenic or benign impact (criteria PS3 and BS3, respectively) and can be used as evidence type to reach a final classification. Moreover, the Clinical Genome Resource Sequence Variant Interpretation Working Group developed rule specifications to refine the PS3/BS3 criteria. Recently, Lira PC et al. developed the “Hi Set” approach that generated PS3/BS3 codes for over two-thousands *BRCA1* VUS. While highly successful, this approach did not discriminate a group of variants with conflicting evidences. Here, we aimed to implement the outcomes of the “Hi-set” approach applying Green Fluorescent Protein (GFP)-reassembly assays, assessing the effect of variants in the RING and BRCT domains of BRCA1 on the binding of these domains with the UbcH5a or ABRAXAS proteins, respectively. The analyses of 26 clinically classified variants, including 13 tested in our previous study, showed 100% sensitivity and specificity in identifying pathogenic and benign variants for both the RING/UbcH5a and the BRCTs/ABRAXAS interactions. We derived the strength of evidences generated by the GFP-reassembly assays corresponding to moderate for both PS3 and BS3 criteria assessment. The GFP-reassembly assays were applied to the functional characterization of 8 discordant variants from the study by Lyra et al. The outcomes of these analyses, combined with those reported in the “Hi Set” study, allowed the assignment of ACMG/AMP criteria in favor or against pathogenicity for all 8 examined variants. The above findings were validated with a semi-quantitative Mammalian Two-Hybrid approach, and totally concordant results were observed. Our data contributes in shedding light on the functional significance of *BRCA1* VUS and on their clinical interpretation within the ACMG/AMP framework.

## 1 Introduction

Germline pathogenic variants (PVs) in the tumor suppressor genes *BRCA1* (MIM#113705) and *BRCA2* (MIM#600185) confer a cumulative lifetime risk of developing breast cancer (BC) by age 80 of 72% and 69%, respectively (1). Sequencing of these genes has become a crucial step of the clinical management of families affected with Hereditary Breast/ovarian Cancer (HBOC) as the carriers of PVs may be addressed to appropriate surveillance programs or risk reducing options, whereas the non carriers may be advised to follow the same recommendations offered to the general population (2). However, as with other disease related genes, the usefulness of this approach is limited by the occurrence of gene variants of uncertain significance (VUS), whose clinical relevance remains elusive (3). A large fraction of VUS identified in *BRCA1* and *BRCA2* are missense changes whose impact on protein function and cancer risk is unknown. Several of these variants are very rare and due to lack of available genetic, clinical, and pathological data, the multifactorial approaches used for assessment of pathogenicity (4) cannot be applied.

The *BRCA1* and *BRCA2* genes encode large nuclear proteins of 1863 amino acids and 3418 amino acids, respectively. BRCA1 contains two well-defined functional domains, the amino terminal Really Interesting New Gene (RING) and the tandem BRCA1 carboxy-terminal repeats (BRCTs). BRCA2 is essentially composed by a DNA-binding domain (DBD) and eight conserved motifs, termed BRC repeats (5, 6). Through these domains, BRCA1 and BRCA2 form protein complexes involved in essential biological processes, such as the maintenance of the genomic integrity *via* homologous recombination (HR)-mediated DNA repair, the regulation of the oxidative stress and protein stability, and the modulation of gene transcription and cell cycle progression (7–13). Therefore, defective protein-protein interactions (PPIs) may lead to uncontrolled cell replication and genomic instability underlying *BRCA1* and *BRCA2* associated tumors (14, 15). In particular, the PPI network of BRCA1 include the E2 ligase UbcH5a (MIM# 602961) which is engaged with BRCA1/BARD1 heterodimer (16) to preferentially catalyze atypical, non degradative K6-linked polyubiquitination related to DNA replication and repair (17–20), and ABRAXAS (MIM# 611143) (21–23) which contributes to BRCA1-dependent DNA damage responses by mediating its recruitment to sites of DNA damage (22, 24, 25). Concerning BRCA2, DSS1 (MIM# 601285), a highly acidic 70 residue polypeptide, associates with a portion of the BRCA2 DBD encompassing the HD and the OB1 and OB2 motifs (6). Recently, it was shown that cancer-causing missense mutations mapped to the HD and OB1 motifs affect the binding with DSS1 allowing mis-localization of BRCA2 to the cytoplasm and defective HR in DNA repair process (26). These observations emphasize that functional assays evaluating the effect of VUS on PPIs may provide helpful clues on their pathogenicity. Indeed, in a previous study, we developed highly accurate gene region-specific functional assays based on the *in vitro* Green Fluorescent Protein (GFP)-reassembly technique and showed that the ascertainment of the effect of *BRCA1* and *BRCA2* VUS on the binding with UbcH5a and DSS1, respectively, correlate with prediction of pathogenicity (27).

The guidelines for variant interpretation of the American College of Medical Genetics and Genomics along with the Association for Molecular Pathology (ACMG/AMP) state that “well-established” functional studies provide evidence of a pathogenic or benign impact (criteria PS3 and BS3, respectively) and can be used to reach a final classification (28, 29). Recently, Lyra PC Jr et al. developed a specific process, designed “Hi Set” approach, to generate ACMG/AMP evidence criteria in favor or against pathogenicity, based on the outcomes of functional analyses (30). This approach relies on the integration of results from a set of 22 well-established functional assays fulfilling the following recommendations: *a)* test more than 10 variants, *b)* test at least 4 benign and 4 pathogenic controls (classified according to the IARC 5-class model), and *c)* achieve a specificity and sensitivity ≥80%. Based on this method, for variants with conflicting results between different functional assays, named discordant variants, a ratio between functional (ben) and non functional (path) results of 3:1 or greater and 1:3 or smaller (1.5≤ log_2_ Ben/Path ≤-1.5) constitutes preponderance of BS3 or PS3 evidence, respectively. In the study, the PS3 and BS3 evidence were assigned to 2355 of 2449 functionally assessed *BRCA1* missense variants recorded, as of August 2019, in the Functional AssaY Integration for BRCA1 (FYI-BRCA1) data set (URL: http://iscva.moffitt.org/fyi-hboc/build/). While highly successful, this approach did not discriminate a group of discordant variants (n=94) for which further analysis are necessary.

In the current study, we aimed to generate additional helpful evidence to resolve the interpretation of eight selected discordant variants mapped to the RING finger and BRCT domains of BRCA1. We evaluated how these variants interfere with the binding of the two domains with UbcH5a or ABRAXAS, respectively, by applying the previously established *in vitro* GFP-reassembly screening. We verified the performance of the BRCA1-ABRAXAS binding assay in correctly discriminating between pathogenic and non-pathogenic variants, analyzing a panel of variants classified according to the IARC 5-class model (Leiden Open Variation Database, URL: http://hci-exlovd.hci.utah.edu/variants.php) (31). In addition, we validated the data obtained from the GFP-reassembly screening with an *in vivo* semi-quantitative Mammalian Two-Hybrid (M2H) approach, as an alternative PPI assay. Finally, we combined the outcomes of these assays with those reported in the “Hi Set” study to assign PS3/BS3 criteria.

## 2 Materials and methods

### 2.1 Cell lines

MCF7, HeLa and HEK293 cell lines were cultured in Dulbecco’s modified Eagle’s medium (DMEM) (Gibco^®^, Life Technologies, Carlsbad, CA, USA) supplemented with 10% heat inactivated Fetal Bovine Serum (Euroclone, Italy) and 1% penicillin/streptomycin (Lonza, Biowhittaker) and maintained in a humidified chamber at 37°C and 5% CO2. The cell lines present in this study were obtained from ATCC^®^ repository (URL: https://www.atcc.org/. MCF7, ATCC^®^ HTB-22; HeLa, ATCC^®^ CCL-2 and HEK293, ATCC^®^ CRL-1573).

### 2.2 Plasmids construction and site-directed mutagenesis

The pET11a-NfrGFP-Z, pMRBAD-Z-CfrGFP and pMRBAD-BRCA1 (amino acids 1-109)-CfrGFP expression vectors (32), were kindly provided by TJ Magliery from the Ohio State University in Columbus (OH, USA). The pET11a-NfrGFP-UbcH5a was generated as described in our previous study (27).

The plasmids pM-GAL4-DBD, pVP16-AD, pM-pVP16, pM-53, pVP16-T, pVP16-CP and pG5-SEAP were included in the Matchmaker Mammalian Assay kit 2 purchased from Clontech (Mountain View, CA, USA).

Full-length *ABRAXAS* cDNA and *BRCA1* cDNA fragment encoding the BRCT domains (amino acids 1646-1863) were synthesized by RT-PCR of total RNA purified from HEK293 cells and cloned into pET11a-NfrGFP-Z between *XhoI* and *BamHI* restriction sites (BRCT domains) or pMRBAD-Z-CfrGFP between *NcoI* and *AatII* restriction sites (ABRAXAS), replacing the fragments encoding leucine zipper (Z) motifs.

The cDNA encoding full-length *UbcH5a* and the cDNA fragments corresponding to *ABRAXAS* amino acids 200-409, *BRCA1* amino acids 1-109 and *BRCA1* amino acids 1643-1863 were generated by PCR using appropriate primers and pMRBAD-ABRAXAS-CfrGFP, pMRBAD-UbcH5a-CfrGFP, pMRBAD-BRCA1 (amino acids 1-109)-CfrGFP or pET11a-NfrGFP-BRCA1 (amino acids 1646-1863) as template, followed by cloning into pVP16-AD (ABRAXAS, UbcH5a, *EcoRI/HindIII* or *EcoRI/BamHI* restriction sites) or pM-GAL4-DBD (BRCA1, *EcoRI/BamHI* restriction sites) vectors (Clontech, Mountain View, CA, USA).

The selected BRCA1 mutations were introduced by PCR-mediated directed mutagenesis of pMRBAD-BRCA1 (amino acids 1-109/1643-1863)-CfrGFP or pM-GAL4-DBD-BRCA1 (amino acids 1-109/1643-1863) using the QuickChange XL Site-directed Mutagenesis Kit (Agilent, Santa Clara, CA, USA) according to the manufacturer’s instructions.

All the plasmids were subjected to DNA sequencing (Eurofins Genomics, Ebersberg, Germany) to confirm the correct reading frame, the presence of the variant to be tested, and the absence of additional mutations introduced during PCR amplification. The oligonucleotides used for generating all the mutant constructs were synthesized by Eurofins Genomics (Ebersberg, Germany) and are listed in **Supplemental Table 1**.

### 2.3 GFP-fragment reassembly screening

Plasmid DNA were isolated using GeneJet plasmid miniprep Kit (Thermo Fisher, Vilnius, Lithuania) according to manufacturer’s instructions. ArcticExpress (D3) *E. coli* competent cells (Agilent, Santa Clara, CA, USA) were co-transformed by heat-shock method with the following compatible pairs of plasmids: *a)* pET11a-NfrGFP-UbcH5a and pMRBAD-BRCA1 (amino acids 1-109)-CfrGFP (both as wild-type or mutant forms), *b)* pMRBAD-ABRAXAS-CfrGFP and pET11a-NfrGFP-BRCA1 (amino acids 1646-1863, both as wild-type or mutant forms), *c)* non-cognate negative controls pET11a-NfrGFP-UbcH5a/pMRBAD-Z-CfrGFP or pET11a-NfrGFP-Z/pMRBAD-ABRAXAS-CfrGFP, and screened for the occurrence of the GFP-fragment reassembly, as previously described (27). ArcticExpress (D3) *E.coli* cells were co-transformed twice with each compatible pair of plasmids. Fluorescence images were captured after excitation with long-wave (365nm) UV light using Azure 600 Imaging System (Dublin, CA, United States) as specified by the manufacturer. All pictures were taken with the same setting of instrument.

### 2.4 Mammalian Two-Hybrid assay

This assay exploits the modular nature of eukaryotic transcription factors composed of two physically and functionally separable domains, a DNA-binding domain (BD) and a transcription activation domain (AD). In particular, the BD of galactose-gene activator GAL4 and the AD derived from the VP16 protein of herpes simplex virus, are individually fused to two interacting protein domains. The reconstitution of the transcription factor – tethered by the interaction between two peptides - is detected by the transcriptional activation of a reporter gene, whose protein product amount can be used as a direct measure of PPI. As reporter vector, we used the pG5-SEAP which encodes the secreted alkaline phosphatase (SEAP). The SEAP is a truncated form of the placental enzyme without the membrane anchoring domain and can be assayed by sampling the culture medium, avoids the need for cell lysis. Levels of SEAP activity detected are directly proportional to changes in intracellular concentrations of SEAP protein (33, 34).

The M2H screening were set up using the Matchmaker Mammalian Assay Kit 2 (Clontech, Mountain View, CA, USA) that provides the following set of vectors: pG5-SEAP encoding the reporter SEAP protein, pM encoding the unfused GAL4 DNA-binding domain (BD), pVP16 encoding the unfused transcription activation domain (AD), pM-pVP16 encoding a fusion peptide of the GAL4 DNA-BD to the VP16 AD that constitutively activates SEAP transcription, pM-53 encoding a fusion peptide of the GAL4 DNA-BD to the mouse p53 protein, pVP16-T encoding a fusion protein of the VP16 AD to the SV40 large T-antigen, which is known to interact with p53, pVP16-CP encoding a fusion peptide of the VP16 AD to the viral coat protein (CP), which does not interact with p53.

The pM and pVP16 vectors were used to generate fusion peptides of GAL4 DNA-BD to BRCA1 RING or BRCT domains (both as wt and mutant forms) and fusion peptides of VP16 AD to UbcH5a or ABRAXAS (aa 200-409), respectively.

Plasmid DNA were isolated using PureLink™ HiPure Plasmid Miniprep Kit (Thermo Fisher, Vilnius, Lithuania) according to manufacturer’s instructions. MCF7, HeLa and HEK293 cells were co-transfected with the pG5-SEAP reporter vector along different plasmid DNA combination using Lipofectamine™ LTX Reagent with PLUS™ Reagent (Thermo Fisher, USA), in Opti-MEM™ I Reduced-Serum Medium (Gibco^®^, Life Technologies, Carlsbad, CA, USA), as specified by the manufacturer. Each cell line was co-transfected in triplicate. After 48 hours, 50µl of culture media of each co-transfection were harvested to discard cell debris, then subjected to SEAP levels quantification using the GreatEscAPe™ SEAP Chemiluminescence Detection Kit (Clontech, Mountain View, CA, USA) according to manufacturer’s instructions. Results were reported in bar graphs (GraphPad Prism software version 5.0) depicting the mean of relative SEAP luminescence ± (standard error of the mean) (SEM) of the 3 biological replicates.

### 2.5 Western blotting

Co-transformed ArcticExpress (D3) *E. coli* cells were recovered from inducing (20µM IPTG and 0.2% L-arabinose) LB agar plates and resuspended in 1ml of 1X phosphate buffered saline (PBS). After centrifugation, each pellet were resuspended in 500µL of B-PER™ II Bacterial Protein Extraction Reagent (Thermo Fisher Scientific, Rockford, IL, USA), supplemented with 2µL lysozyme (Euroclone, Italy), 1µL DNase I (Promega, Italy) and protease inhibitor EDTA-free (Thermo Fisher Scientific, Eugene, Oregon, USA), to extract soluble proteins following the manufacturer’s instructions. The protein concentration of the whole cell extracts was determined by the Bradford method using the Bio-Rad protein assay kit (Bio-Rad Laboratories, Hercules, CA, USA) according to manufacturer’s instructions. Equal amounts of protein (20µg) were subjected to 4–20% precast gradient polyacrylamide gel (Bio-Rad Laboratories, Hercules, CA, USA) and visualized by Western blotting using anti-GFP antibody.

Transfected HEK293 cells were harvested and resuspendend in 300µL of RIPA lysis buffer (50mM Tris-HCL pH 7.5, 150mM NaCl, 1mM EDTA, 1% (v/v) Triton-X100, 1% (w/v) sodium deoxycholate, 0.1% (w/v) SDS) supplemented with protease inhibitor EDTA-free (Thermo Fisher Scientific, Eugene, Oregon, USA). After sonication, cell debris were removed by centrifugation at 13000 rpm at 4°C for 15 minutes and the supernatants were collected as soluble proteins fraction. The protein concentration was determined by the Bradford method using the Bio-Rad protein assay kit (Bio-Rad Laboratories, Hercules, CA, USA) according to manufacturer’s instructions. Equal amounts of protein (50µg) were subjected to 4–20% precast gradient polyacrylamide gel (Bio-Rad Laboratories, Hercules, CA, USA) and visualized by Western blotting using specific antibodies. Protein signals were captured on a X-ray film, or by employing Azure 600 Imaging System (Dublin, CA, United States) as specified by the manufacturer. The antibodies used in this study were listed in **supplemental table 2**.

### 2.6 *In silico* data

The prior probability of pathogenicity of the selected variants were derived from the BRCA Exchange website (https://brcaexchange.org/). The *In Silico* Prior Probability of Pathogenicity accounts for impact on protein functions or interference with mRNA splicing. Accordingly to default parameter values, we selected variants with protein level estimation ≥0.66 (http://priors.hci.utah.edu/PRIORS/) or ≥0.28 (BayesDel, https://fengbj-laboratory.org/BayesDel/BayesDel.html) corresponding to moderate/high prior probability of pathogenicity, and splicing-level estimation ≤0.04 (http://priors.hci.utah.edu/PRIORS/) corresponding to low probalility to be spliceogenic.

## 3 Results

### 3.1 BRCA1 discordant variants selection

**Figure 1:**
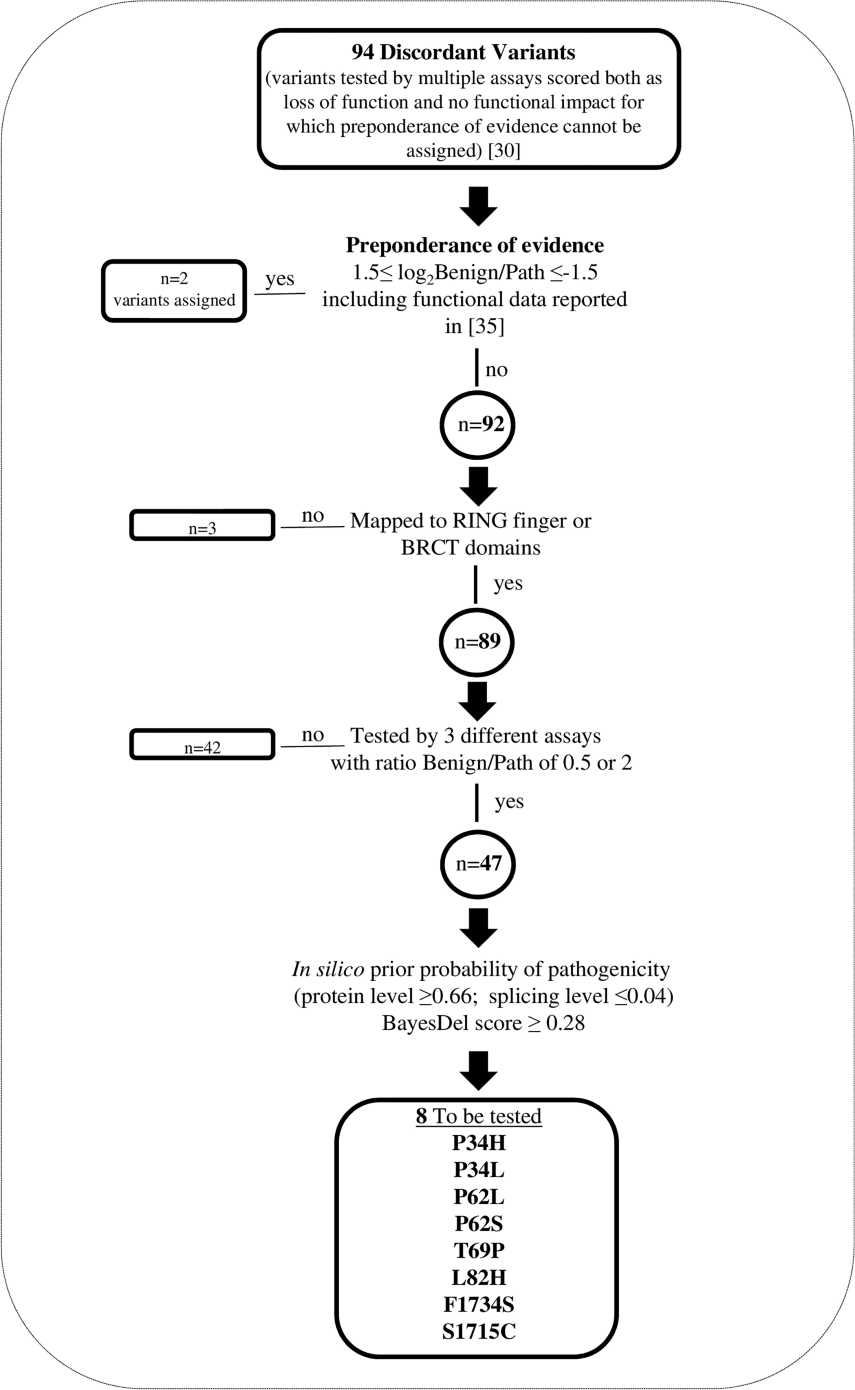
Selection of *BRCA1* discordant variants tested by GFP-reassembly and M2H assays. Flow chart outlining the selection process.

We focused on the group of the 94 discordant variants for which, by applying the “Hi Set” approach (30), preponderance of BS3 or PS3 evidence cannot be assigned. Based on the process illustrated in **Figure 1**, we selected 8 of the 94 discordant variants, mapped to the gene regions encoding RING or BRCT domains, as follows. Firstly, we included into the “Hi Set” process for the assignment of preponderance of evidence the data from 3 additional functional assays (35), published subsequently to the last update (August 2019), of the FYI-BRCA1 data set considered in the article by Lyra PC Jr et al. (30) and fulfilling the criteria described above. This allowed to assign BS3 and PS3 evidence to the p.Met1400Val and p.Glu1735Lys variants, respectively. Among the remaining 92 variants, we selected those mapped to the RING finger or BRCT domains (n=89). Then, we selected those classified as discordant based on the results of 3 different assays (n=47), and with a ratio between the benign and pathogenic observations of 0,5 or 2, i.e. variants for which the addition of the results of a single assay would provide evidence for preponderance of PS3 or BS3 criteria based on the “Hi Set” process. Among the above 47 variants, we further selected those (n=8) with moderate/high prior probability of pathogenicity and low prior probability to be spliceogenic, estimated by in *silico* analyses (HCI Breast Cancer Genes Prior Probabilities, http://priors.hci.utah.edu/PRIORS/; BayesDel, https://fengbj-laboratory.org/BayesDel/BayesDel.html). These variants, including p.Pro34His, p.Pro34Leu, p.Pro62Leu, p.Pro62Ser, p.Thr69Pro, p.Leu82His, p.Phe1734Ser and p.Ser1715Cys, were tested by the GFP-reassembly and M2H assays (**Supplementary Table 3**).

### 3.2 Functional evaluation of BRCA1 discordant variants by GFP-reassembly assay

Based on the results observed for a validation panel of 11 pathogenic (class 4 and 5) and 2 benign (class 1) variants, we previously shown that the GFP-reassembly assay analyzing the effect of *BRCA1* VUS mapped to the RING domain on the binding with UbcH5a has 100% specificity and sensitivity (27).

**Figure 2:**
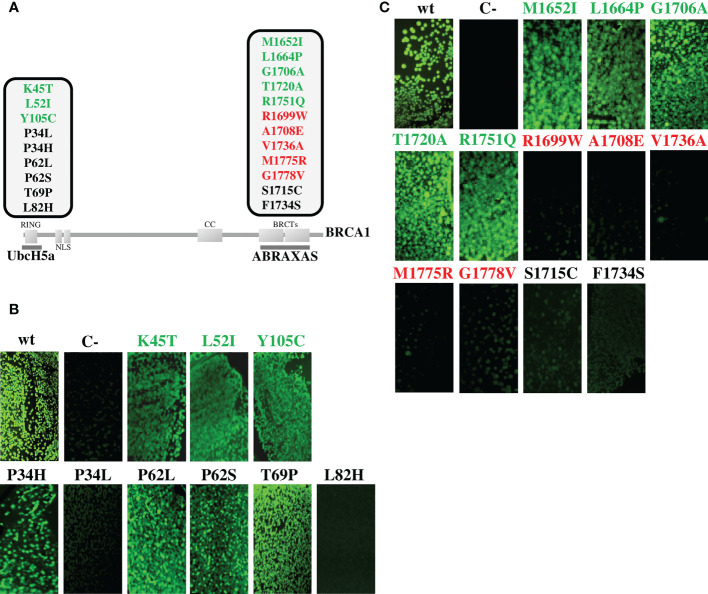
Functional evaluation of *BRCA1* variants by GFP-reassembly *in vitro* assay. **(A)** Schematic representation of the BRCA1 protein showing functional domains, interacting proteins and variants analyzed in this study. The benign variants (class 1) are shown in green, the pathogenic variants (class 5) are shown in red and the discordant variants are shown in black. The partner proteins are depicted underneath their respective BRCA1 interacting domains. **(B)** Detection of BRCA1-UbcH5a binding by GFP-reassembly *in vitro* assay. Wild-type NfrGFP-UbcH5a/BRCA1-CfrGFP peptides (wt) and non-cognate NfrGFP-UbcH5a/Z-CfrGFP peptides (C-) were included as internal positive and negative control, respectively. **(C)** Detection of BRCA1-ABRAXAS binding by GFP-reassembly *in vitro* assay. Wild-type NfrGFP-ABRAXS/BRCA1-CfrGFP peptides (wt) and non-cognate NfrGFP-Z/ABRAXAS-CfrGFP peptides (C-) were included as internal positive and negative control, respectively. Fluorescence images were captured after excitation with long-wave (365nm) UV light. Arctic Express (D3) *E.coli* bacterial cells were co-transformed twice with each compatible pair of plasmids.

In the current study, to meet the selection criteria of the “Hi Set” approach, we expanded the validation panel including 3 additional benign variants mapped to the RING domain, and 5 pathogenic and 5 benign variants mapped to the BRCT domains (**Figure 2A**, **Table 1**) (4). All these mutant constructs were co-transformed in bacterial cells with the pET11a-NfrGFP-UbcH5a or pMRBAD-ABRAXAS-CfrGFP expressing full length UbcH5a or ABRAXAS wild type proteins. Under inducing conditions, a bright fluorescence, as a result of PPI, was observed in bacterial cells co-expressing UbcH5a or ABRAXAS together with BRCA1 bearing all benign (class1) variants. Conversely, no fluorescence was observed in bacterial cells when UbcH5a or ABRAXAS were co-expressed with all the pathogenic variants. These results indicate 100% specificity and sensitivity of both assays (**Figures 2B, C**).

**Table 1:** Variants of validation panel.

Validation panel [Leiden Open Variation Database, URL: http://hci-exlovd.hci.utah.edu/variants.php]							
Domain	DNA change	Protein change	IARC class^a^	Domain	DNA change	Protein change	IARC class^a^
**RING Finger**	c.53T>C	p.Met18Thr^b^	**4**	**BRCTs**	c.4956G>A	p. Met1652Ile^c^	**1**
	c.65T>C	p.Leu22Ser^b^	**5**		c.4991T>C	p.Leu1664Pro^c^	**1**
	c.110C>A	p.Thr37Lys^b^	**5**		c.5117G>C	p.Gly1706Ala^c^	**1**
	c.115T>C	p.Cys39Arg^b^	**5**		c.5158A>G	p.Thr1720Ala^c^	**1**
	c.116G>A	p.Cys39Tyr^b^	**5**		c.5252G>A	p.Arg1751Gln^c^	**1**
	c.122A>G	p.His41Arg^b^	**5**		c.5095C>T	p.Arg1699Trp^c^	**5**
	c.130T>A	p.Cys44Ser^b^	**5**		c.5123C>A	p.Ala1708Glu^c^	**5**
	c.131G>T	p.Cys44Phe^b^	**5**		c.5207T>C	p.Val1736Ala^c^	**5**
	c.131G>A	p.Cys44Tyr^b^	**5**		c.5324T>G	p.Met1775Arg^c^	**5**
	c.181T>G	p.Cys61Gly^b^	**5**		c.5363G>T	p.Gly1788Val^c^	**5**
	c.191G>A	p.Cys64Tyr^b^	**5**				
	c.133A>C	p.Lys45Gln^b^	**1**				
	c.134A>C	p.Lys45Thr^c^	**1**				
	c.154C>A	p.Leu52Ile^c^	**1**				
	c.199G>T	p.Asp67Tyr^b^	**1**				
	c.314A>G	p.Tyr105Cys^c^	**1**				

a Class 1 benign variants. Class 4/5, likely pathogenic/pathogenic variants, respectively, are indicated in bold.

b Variants included in the previous study (27).

c Variants included in this study.

Four of the discordant variants (p.Pro34His, p.Pro62Leu/Ser and p.Thr69Pro) did not affect interaction of the BRCA1 constructs with the binding protein constructs, while the remaining four variants (p.Pro34Leu, p.Leu82His, p.Ser1715Cys and p.Phe1734Ser) abolished interaction (**Figures 2B, C**).

The expression level of all the peptides was verified by Western blotting analysis using the anti-GFP antibody and no significant differences were observed indicating that the loss of fluorescence observed in the GFP-reassembly *in vitro* assay, was attributable to the lack of binding between the proteins and not to poor expression of the mutant constructs (**Supplemental Figure 1**).

### 3.3 Functional evaluation of BRCA1 discordant variants by Mammalian Two-Hybrid

The data obtained from the GFP-reassembly *in vitro* screening were validated with an *in vivo* semi-quantitative M2H assay.

To set up the optimal experimental conditions, we first tested three different cell lines: *i)* MCF7-; *ii)* HeLa and *iii)* HEK293. In the presence of the pM-pVP16 activator, detectable levels of SEAP reporter expression were observed only when the HEK293 were used as recipient cells (**Supplemental Figure 2A**). Subsequently, the feasibility of the M2H assay cells to detect PPIs in the HEK293 was verified by co-transfecting the cells with the pM-53 and pVP16-T constructs, carrying the coding sequences of the p53 protein and of its interactor SV40 large T-antigen, fused with the GAL4-BD and the VP16-AD, respectively. The observed SEAP expression level was comparable to that observed in the presence of the pM-pVP16 activator, whereas a markedly reduced SEAP expression was detected when the pM-53 construct was co-transfected with a construct carrying the non-interacting polyoma virus coat protein (pVP16-CP) (**Supplementary Figure 2B**).

**Figure 3:**
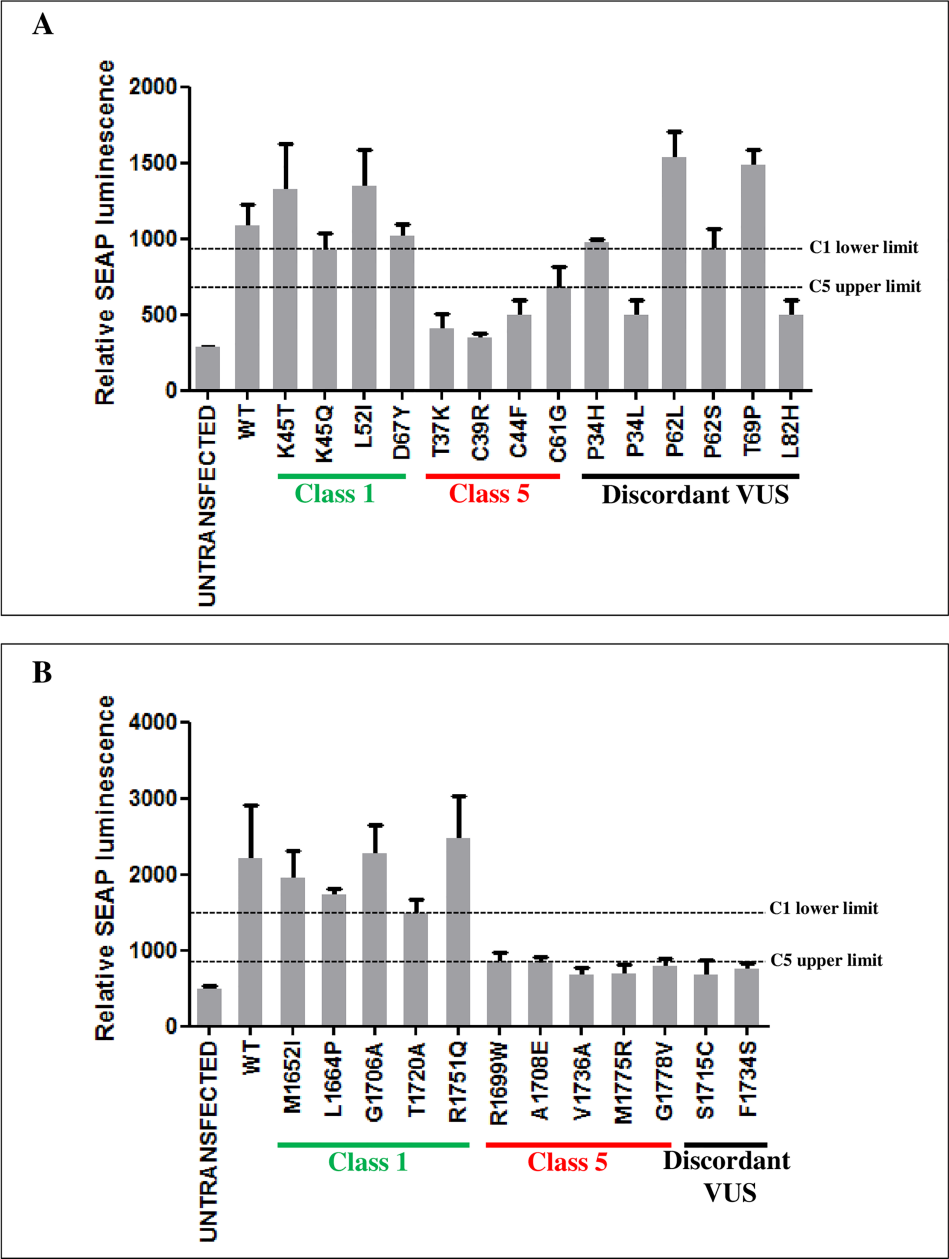
Functional evaluation of *BRCA1* variants by M2H assay. The interaction strength of the BRCA1 RING domain with UbcH5a **(A)** and the BRCA1 BRCT domains with ABRAXAS **(B)**, in the presence of the indicated *BRCA1* variants, was measured as SEAP expression levels. Each cell line was co-transfected in triplicate. The columns show the mean values of the three biological replicates. SEAP expression was evaluated 48h after transfection. Horizontal dotted lines represent the lowest relative SEAP luminescence level of class 1 variants (C1 lower limit) and the highest relative SEAP luminescence level of class 5 variants (C5 upper limit).

Then, to verify the performance of M2H assay in discriminating pathogenic and benign variants, the HEK293 cells were transiently co-transfected with the pG5-SEAP reporter vector along the pVP16 fused to the UbcH5a or ABRAXAS wild type proteins and pM fused to the RING or BRCT domains carrying a subset of the *BRCA1* benign and pathogenic variants included in the validation panel tested in the GFP-reassembly assay. The results shown in **Figure 3** indicate 100% specificity and sensitivity of both assays. Indeed, the distribution of the expression level scores of SEAP, measured as relative luminescence units, indicated high accuracy of both assays, being the average level of the benign variants approximately 1.9 (RING variants) or 2.6 (BRCT variants) times higher than that of the pathogenic variants, with no overlap between the ranges observed for the two sets of variants (**Supplemental Table 4**).

The results of the screening assessing the effect of the discordant variants on the binding with UbcH5a or ABRAXAS were consistent with those of the GFP-reassembly assay. In fact, the p.Pro34Leu, p.Leu82His, p.Ser1715Cys and p.Phe1734Ser variants were observed to mediate expression levels of the reported gene similar to those of the pathogenic variants of the reference set, while the p.Pro34His, p.Pro62Leu/Ser variants and p.Thr69Pro behaved similarly to BRCA1 wild type and benign variants of the reference set (**Figure 3**).

The ectopic protein expression of all peptides in HEK293 transiently transfected cells were confirmed by Western blotting analyses using specific antibodies. No substantial differences were observed between mutagenized and wild type proteins (**Supplemental Figure 3**).

### 3.4 Implementation of the binding assays into the pipeline for assignment of PS3 and BS3 codes

To derive the strength of evidences generated by the GFP-reassembly assays analyzing BRCA1-UbcH5a or BRCA1-ABRAXAS binding, we estimated the Odds of pathogenicity (Oddspath) by applying the formula: OddsPath = [P2 x (1- P1)]/[(1- P2) x P1], according to the Clinical Genome Resource (ClinGen) Sequence Variant Interpretation (SVI) Working Group recommendations (29). The prior probability (P1) represents the proportion of pathogenic variants out of the total number of variant controls tested (11/16 for BRCA1-UbcH5a binding assay, and 5/10 for BRCA1-ABRAXAS binding assay), P2 represents the proportion of pathogenic variants in the groups with functionally abnormal or functionally normal readouts as posterior probabilities. Both functional assays correctly assigned all class 4/5 variants to the functionally abnormal group resulting in the OddsPath of 5 for both BRCA1-UbcH5a and BRCA1-ABRAXAS binding assays. Likewise, all class 1 variants were assigned to the functionally normal group, resulting in the OddsPath of 0.09 for the BRCA1-UbcH5a binding assay and 0.2 for BRCA1-ABRAXAS binding assay (**Supplemental Table 5**). According to the Bayesian adaptation of the ACMG/AMP variant interpretation guidelines (36), these OddsPath correspond to moderate evidence for both pathogenic and benign criteria assessment. Thus, we assigned the PS3_moderate code to *BRCA1* variants which prevented the BRCA1-UbcH5a or BRCA1-ABRAXAS interactions and the BS3_moderate code to those maintaining the binding.

Finally, we combined these outcomes with those reported in the “Hi Set” study. By applying the cutoff 1.5 ≤  log2 Ben/Path ≤ −1.5, we defined preponderance of evidence and assigned as the final evidence the strongest criteria among those available for each variants. In particular, we assigned PS3 code to the p.Pro34Leu, p.Leu82His, p.Ser1715Cys and p.Phe1734Ser and BS3 code to the p.Pro34His, p.Pro62Leu, p.Pro62Ser and p.Thr69Pro BRCA1 variants (**Table 2**).

**Table 2:** Assignment of BS3 and PS3 codes.

Original assignment (30)									Assignment after addition of binding assays [This study]					
Discordant Variants	Functional assays			Count	Sum	Evidence criteria from individual functional assay	Log_2_ B/P	FINAL CALL		Count	Sum	Evidence criteria from individual functional assay	Log_2_ B/P	FINAL CALL
	T131	T133	T134						UbCH5a binding					
**p.Pro34Leu**	1	1	0	3	2	PS3; PS3_moderate; BS3	-1	unassigned	1	4	3	PS3; PS3_moderate; BS3; **PS3_moderate**	**-1.585**	**PS3**
**p.Pro34His**	1	0	0	3	1	PS3; BS3; BS3	1	unassigned	0	4	1	PS3; BS3; BS3; **BS3_moderate**	**1.585**	**BS3**
**p.Pro62Leu**	0	0	1	3	1	BS3; BS3; PS3	1	unassigned	0	4	1	BS3; BS3; PS3; **BS3_moderate**	**1.585**	**BS3**
**p.Pro62Ser**	0	0	1	3	1	BS3; BS3; PS3	1	unassigned	0	4	1	BS3; BS3; PS3; **BS3_moderate**	**1.585**	**BS3**
**p.Thr69Pro**	0	1	0	3	1	BS3; PS3_moderate; BS3	1	unassigned	0	4	1	BS3; PS3_moderate; BS3; **BS3_moderate**	**1.585**	**BS3**
**p.Leu82His**	1	0	1	3	2	PS3; BS3; PS3	-1	unassigned	1	4	3	PS3; BS3; PS3; **PS3_moderate**	**-1.585**	**PS3**
	**T102**	**T115**	**T131**						**ABRAXAS binding**					
**p.Ser1715Cys**	0	1	1	3	2	BS3; PS3; PS3	-1	unassigned	1	4	3	BS3; PS3; PS3; **PS3_moderate**	**-1.585**	**PS3**
	**T27**	**T102**	**T131**											
**p.Phe1734Ser**	0	1	1	3	2	BS3; PS3; PS3	-1	unassigned	1	4	3	BS3; PS3; PS3; **PS3_moderate**	**-1.585**	**PS3**

**T131**, Cell viability assay in HAP1 cells (37); **T133**, BARD1 binding (38); **T134**, E3 activity (38); **T102**, Transcriptional activation - VarCall (39); **T115**, Protease sensitivity (40); **T27**, Phosphopeptide binding activity (40). “**0”**, functionally normal; **“1”**, functionally abnormal. **Count**, Total No of observations. **Sum**, No of pathogenic observations. **B/P**, Ratio of benign to pathogenic observations. BS3/PS3 codes derived from UbcH5a/BRCA1 or ABRAXAS/BRCA1 binding assays are indicated in bold. The log2 of B/P after addition of binding assays data and Final Call are indicated in bold.

## 4 Discussion

About 10-20% of individuals at high risk of HBOC who undergo germline *BRCA1* and *BRCA2* genetic test is found to carry a VUS, whose association with the disease remains elusive (3). The inability to classify a VUS either as disease causing or as rare non-pathogenic variants, represents a roadblock for genetic counseling of carriers, risk evaluation and adoption of preventive and risk-reduction measures. The missense variants constitute the largest class of VUS and their accurate interpretation is further challenged by conflicting functional analysis results, as shown in (30).

Recently, the ClinGen SVI Working Group developed rule specifications to refine the ACMG/AMP PS3 and BS3 criteria, applied to variants affecting or not affecting protein functions, respectively, and defined a four-step process to determine the applicability and strength of evidence of functional assays for the use in clinical variant interpretation (29). According to these recommendations, a series of important considerations have to be applied to functional assays evaluating variant effects. In particular, the appropriateness and robustness of the assay, as well as its ability to reflect the physiological context should be carefully evaluated.

In this respect, experimental analysis assessing the effect of VUS on PPIs can be considered a reliable class of functional assays for the ascertainment of the clinical relevance of variants in genes like *BRCA1*, *BRCA2* and *PALB2* whose protein products elicit their activity through the binding with cellular interactors (41–43). In particular, in this study we examined the proper formation of the BRCA1-UbcH5a and BRCA1-ABRAXAS complexes that are essential for the maintenance of the genomic integrity *via* HR-mediated DNA repair, a key function of *BRCA1* tumor suppressor activity.

In our analysis, the examination of the effect of *BRCA1* VUS on the above mentioned PPIs was based on gene region specific adaptations of the GFP-reassembly assay. One theoretical limitation of this approach is represented by the use of an artificial experimental model, where human proteins, or their functional domains, are expressed in *E.coli* cells. In this environment, it could be argued that the behavior of mutant constructs might not reflect the properties of proteins expressed in their ‘natural’ mammalian context. To address this criticism, we validated the data obtained from the *in vitro* GFP-reassembly screening with an *in vivo* semi-quantitative M2H approach. In this assay, proteins encoded by mammalian cDNAs are more likely to be in their native conformation, and to undergo naturally-occurring post-translational modifications that might be relevant for proper PPIs. Therefore, the outcomes of the M2H assay can serve as a reference to evaluate the accuracy of the GFP-reassembly assay. The analysis of 26 *BRCA1* variants (9 benign, 9 pathogenic and 8 VUS) showed no discordant results between the two approaches, indicating that the binding assay exploiting a non mammalian experimental system was in our hands as accurate as the one performed in mammalian cells. In addition, it has to be noted that, compared to the MH2 assay, the GFP-reassembly assay takes advantage from a greater easiness of use, rapidity and cost-effectiveness. Notably, of the 22 data sets selected in the study by Lyra PC Jr and colleagues (30) for inclusion in the “Hi-set” of well-established functional assays, 7 (32%) exploited *S.cerevisiae* or *E.coli* cells as experimental models (38, 40, 44, 45).

Each of the evidence criteria outlined in the ACMG/AMP sequence variant interpretation guidelines has an assigned weight (supporting, moderate, strong and very strong) to which the evidence can be applied (36). Regarding functional analysis, the higher is the number of clinical validation controls (known benign and known pathogenic variants classified based on multifactorial likelihood models that exclude functional data) tested in the assay considered, and its accuracy in classifying benign and pathogenic variants as functional or non functional, respectively, the strongest is the level of evidence that can be derived from the assay (29). The two BRCA1 region specific GFP-reassembly assays applied in the present study were validated analyzing a set of 26 previously classified pathogenic (n=16) or benign (n=10) variants, including 13 tested in our previous study (27). Of these, 16 (11 pathogenic and 5 benign) mapped to the RING domain and 10 (5 pathogenic and 5 benign) to the BRCT domains. In both assays all control pathogenic variants were observed to abolish PPIs, whereas in the presence of all control benign variants, the occurrence of PPIs was detected. Based on the OddsPath values derived from the above observations, we could assign to both assays a strength evidence corresponding to moderate for both PS3 and BS3 criteria (29).

The GFP-reassembly assays we developed were applied to the analysis of 8 *BRCA1* VUS selected among a set of 94 variants to which, in the integrated analysis by Lyra PC Jr et al. (30), no PS3 or BS3 code could be unambiguously assigned based on the data available at the time of the study. Four variants (p.Pro34His, p.Pro62Leu, p.Pro62Ser and p.Thr69Pro) were observed to be functional (maintenance of PPIs), whereas the remaining 4 (p.Pro34Leu, p.Leu82His, p.Ser1715Cys and p.Phe1734Ser) were classified as non functional (loss of PPIs). Integrating these observations with the information reported in the above mentioned publication (30), a BS3 code could be attributed to all 4 functional variants and a PS3 code to all 4 non-functional variants. Remarkably, all the BS3-coded variants were predicted by in *silico* analyses to be at high or moderate probability of pathogenicity (**Supplementary Table 3**).

Our results yield additional evidences in favor of the reliability of the non-mammalian GFP-reassembly assay in providing functional evidences useful for the clinical classification of VUS. In addition, they emphasize the benefits of integrating additional approaches to resolve, within the ACMG/AMP-based framework model, the interpretation of variants with discordant functional observations and provide further support to the notion that combining results from different assays may increase confidence in the final BS3/PS3 code assignment.

The outcome of this study will contribute to inform the clinical classification of *BRCA1* variants presently classified as VUS. This will lead to an increase in the number of families that can be correctly classified as linked or unlinked to this gene, improving the predictivity and the clinical usefulness of genetic testing for HBOC.

## Data availability statement

The original contributions presented in the study are included in the article/**Supplementary Material**. Further inquiries can be directed to the corresponding author.

## Author contributions

LC conceived the study, performed experimental analyses, analyzed the data, and drafted the manuscript. PR conceived and supervised the study and revised the manuscript. All authors contributed to the article and approved the submitted version.
