# Supplementary figures and images for: Refinement of the assignment to the ACMG/AMP BS3 and PS3 criteria of eight BRCA1 variants of uncertain significance by integrating available functional data with protein interaction assays
Source: Front Oncol. 2023 Apr 24;13:1146604. doi: 10.3389/fonc.2023.1146604 (PMC10164951; doi:10.3389/fonc.2023.1146604)

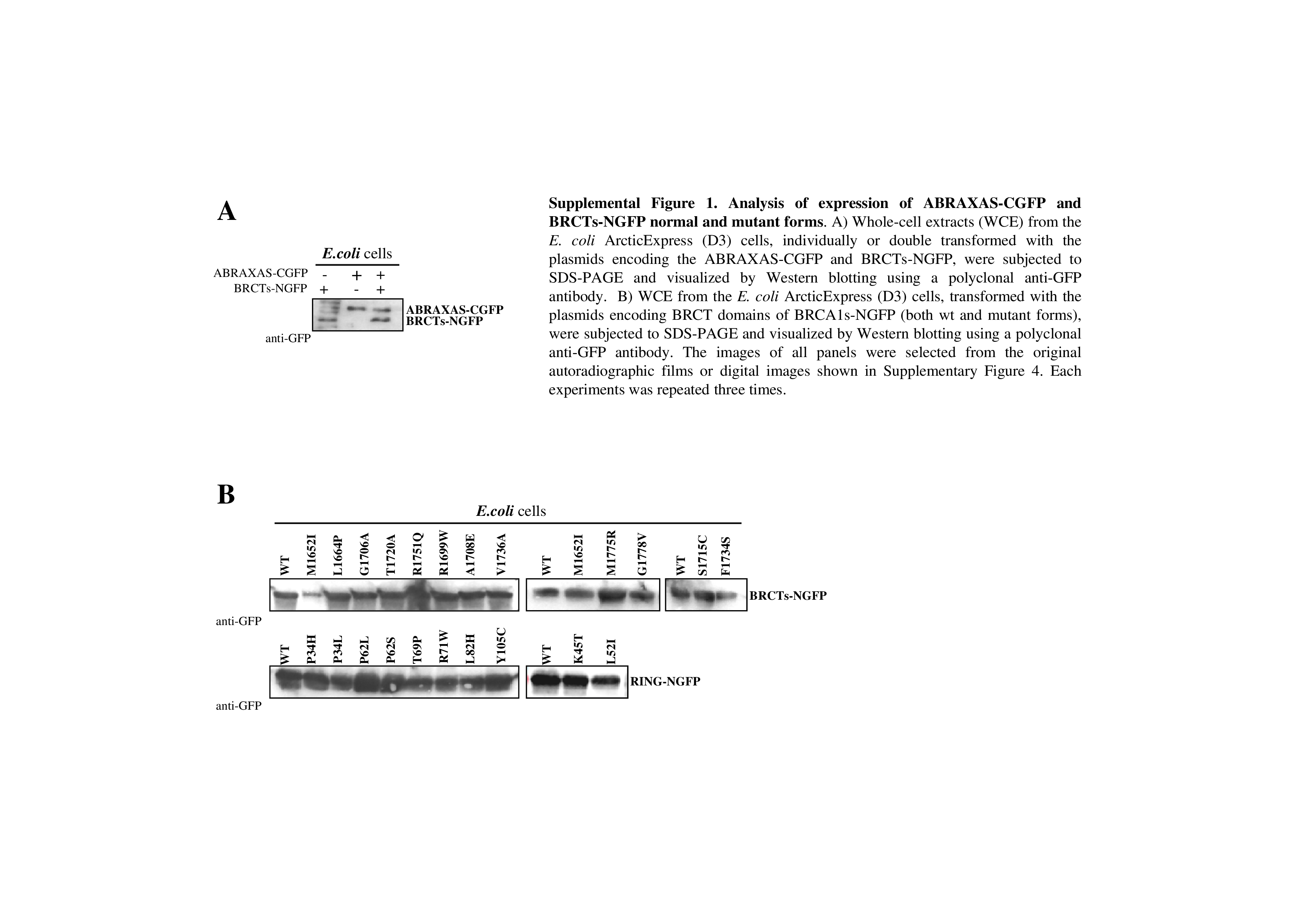

Supplement: Supplementary file 1 [file Image_1.tiff]

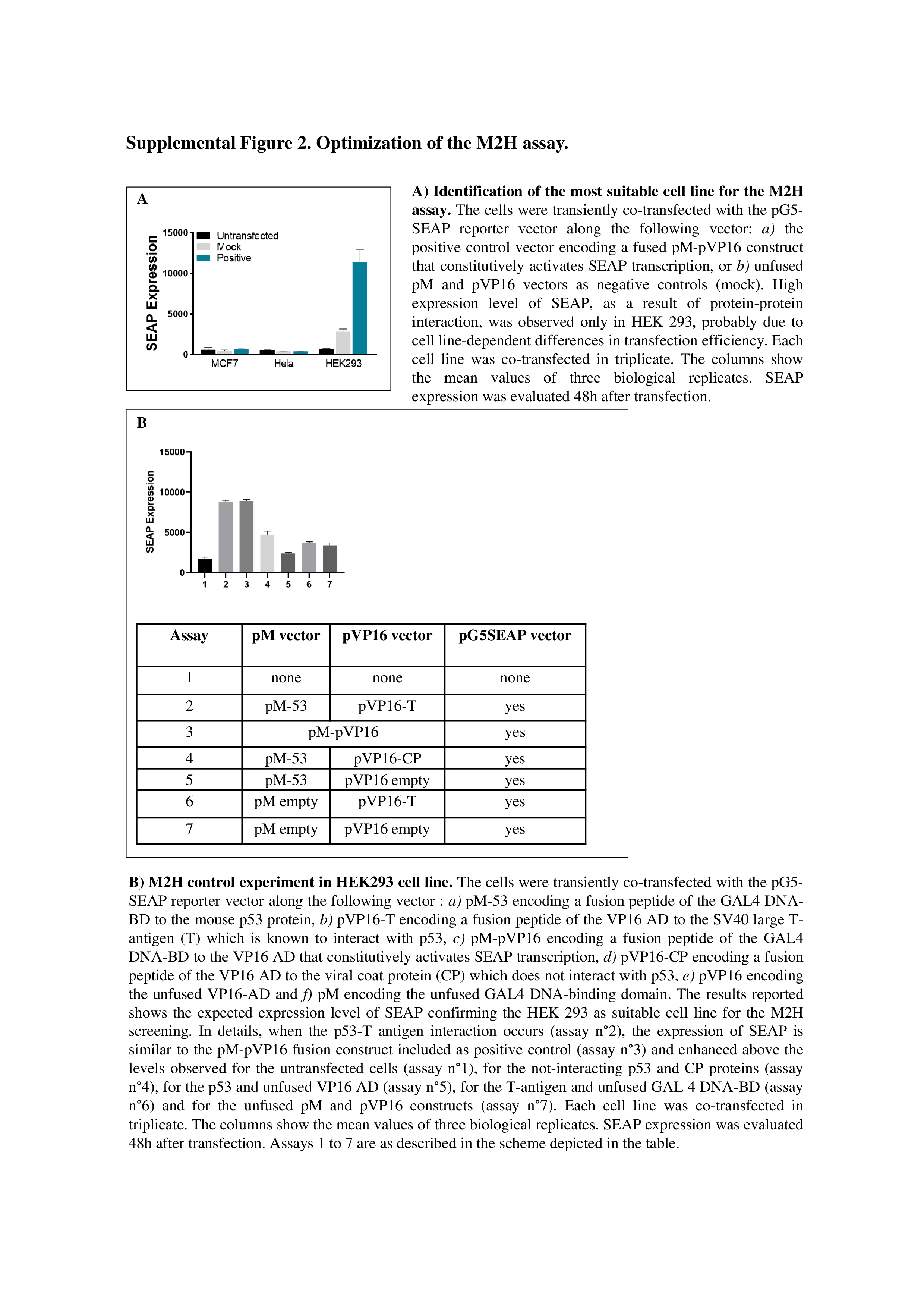

Supplement: Supplementary file 2 [file Image_2.tiff]

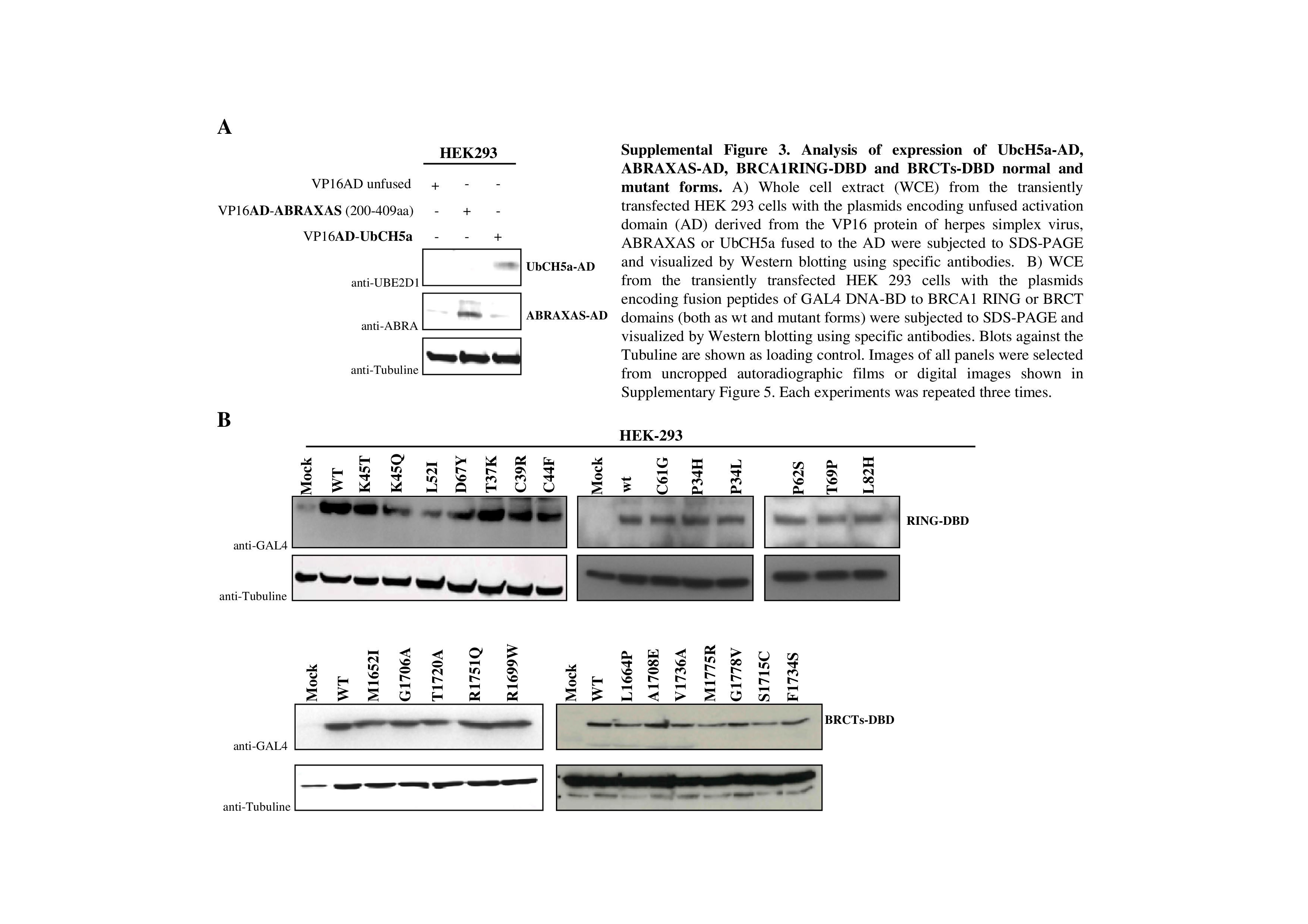

Supplement: Supplementary file 3 [file Image_3.tiff]

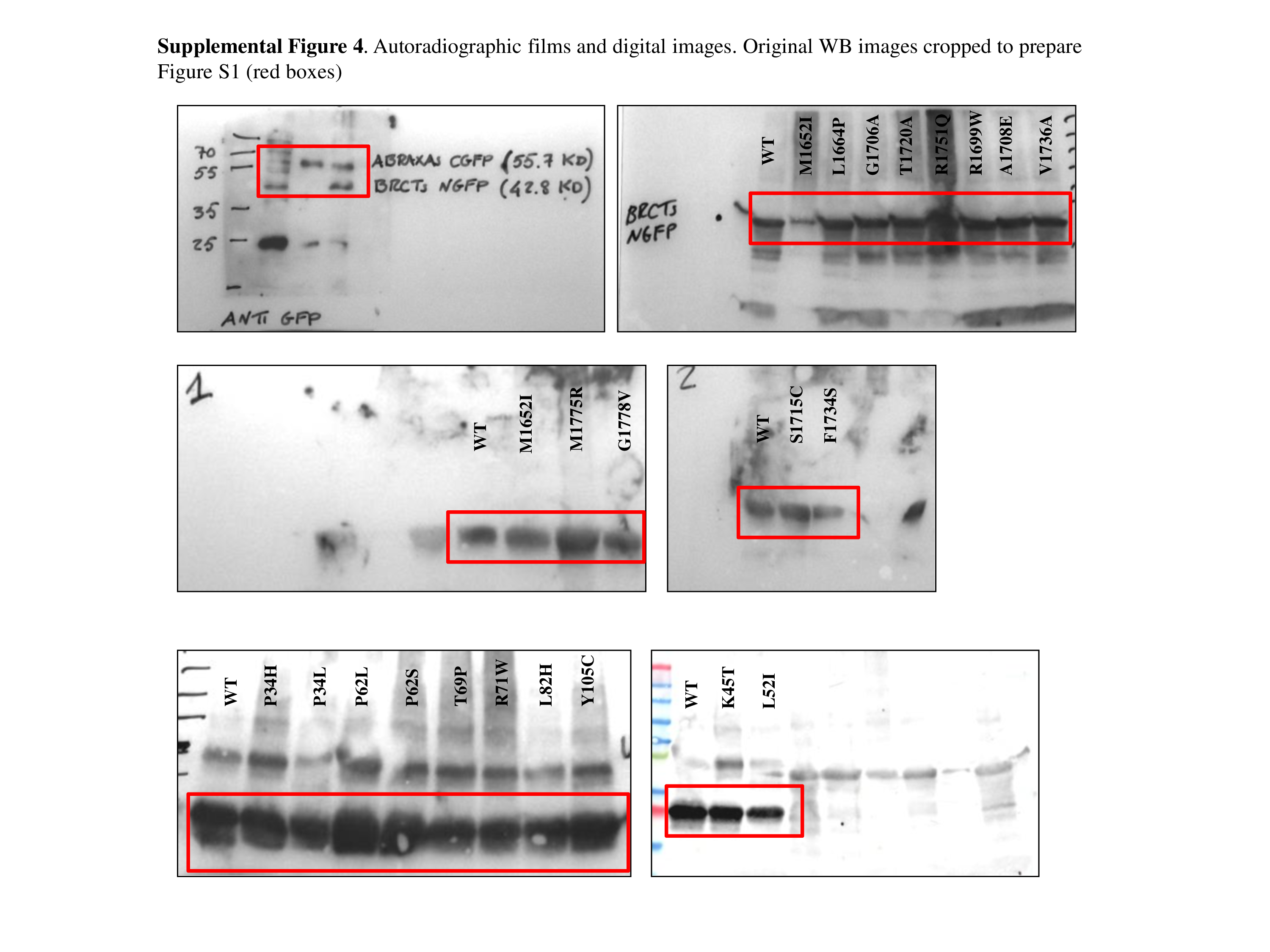

Supplement: Supplementary file 4 [file Image_4.tiff]

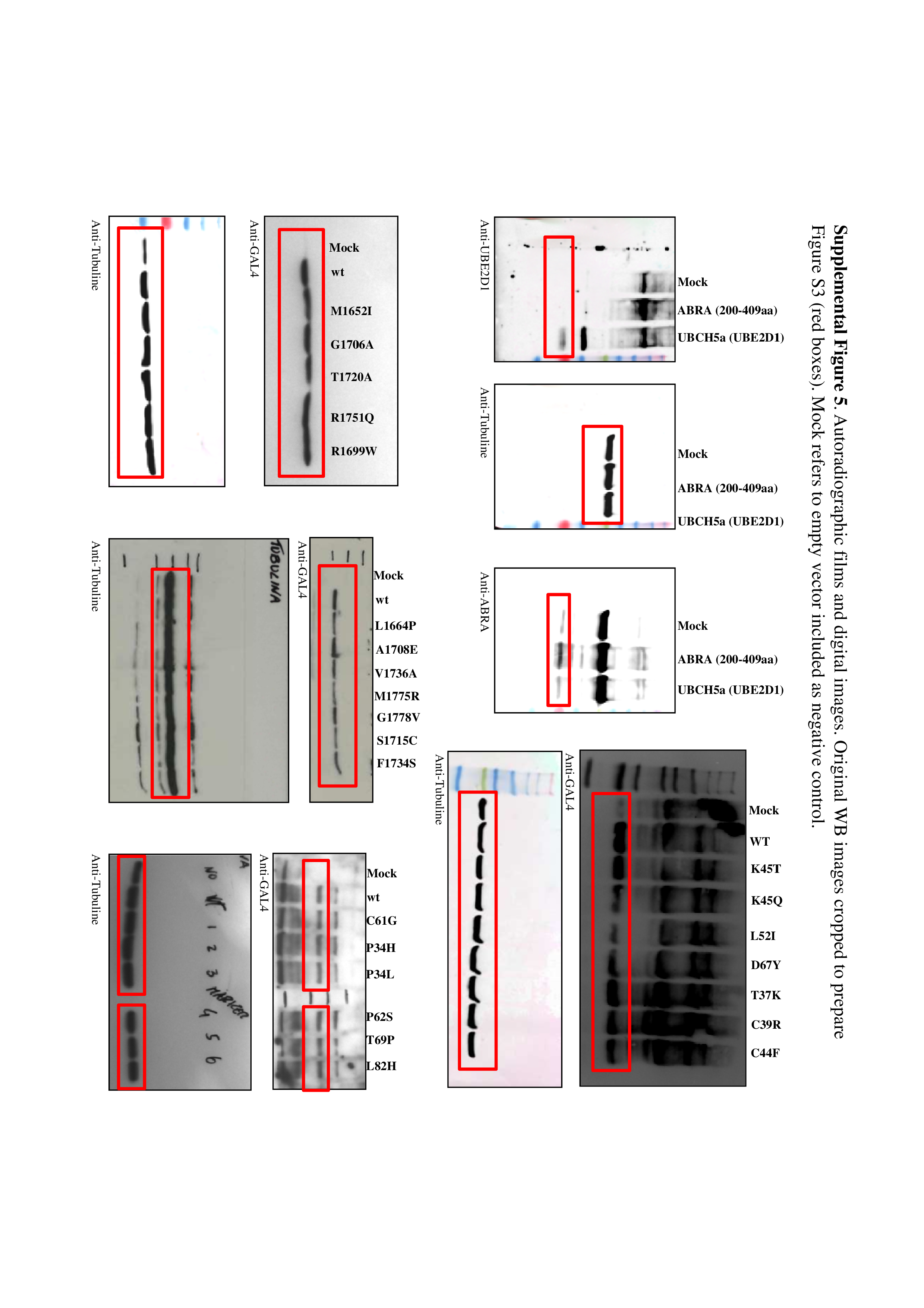

Supplement: Supplementary file 5 [file Image_5.tiff]
